# Quantitative Proteomics Analysis Reveals the Effect of a MarR Family Transcriptional Regulator *AHA_2124* on *Aeromonas hydrophila*

**DOI:** 10.3390/biology12121473

**Published:** 2023-11-28

**Authors:** Zhen Li, Wanxin Li, Jinlian Lu, Ziqiu Liu, Xiangmin Lin, Yanling Liu

**Affiliations:** 1 Zhangzhou Health Vocational College, Zhangzhou 363000, China; lizhenlyx@aliyun.com; 2 Fujian Provincial Key Laboratory of Agroecological Processing and Safety Monitoring, School of Life Sciences, Fujian Agriculture and Forestry University, Fuzhou 350002, China; lujinlian1998@163.com (J.L.); xiangmin@fafu.edu.cn (X.L.); 3 School of Public Health, Fujian Medical University, Fuzhou 350122, China; liwx2012@126.com; 4 Key Laboratory of Marine Biotechnology of Fujian Province, Institute of Oceanology, Fujian Agriculture and Forestry University, Fuzhou 350002, China; 5 National Engineering Research Center of Juncao Technology, Fujian Agriculture and Forestry University, Fuzhou 350002, China

**Keywords:** *Aeromonas hydrophila*, MarR family transcriptional regulator, biological function

## Abstract

**Simple Summary:**

*Aeromonas hydrophila* is a major pathogen with human–animal–fish comorbidity that causes many diseases. To better investigate the roles of different genes in *Aeromonas hydrophila*, our aim is to knock out a gene in *A. hydrophila* and study whether the gene affects the physiological phenotypes or not. We found that *AHA_2124*, encoding a multiple antibiotic resistance regulator (MarR) family regulator, affected the extracellular protease activity, hemolytic activity, and motility of the phytopathogenic bacterium *Aeromonas hydrophila*. The MarR family of the transcriptional regulators of bacteria are mainly involved in the regulation of various cellular processes. In this work, we call for further research on the bioinformatics analysis based on the comparison of the Δ*AHA_2124* strain and wild type.

**Abstract:**

The transcriptional regulators of the MarR family play an important role in diverse bacterial physiologic functions, whereas their effect and intrinsic regulatory mechanism on the aquatic pathogenic bacterium *Aeromonas hydrophila* are, clearly, still unknown. In this study, we firstly constructed a deletion strain of *AHA_2124* (Δ*AHA_2124*) of a MarR family transcriptional regulator in *Aeromonas hydrophila* ATCC 7966 (wild type), and found that the deletion of *AHA_2124* caused significantly enhanced hemolytic activity, extracellular protease activity, and motility when compared with the wild type. The differentially abundant proteins (DAPs) were compared by using data-independent acquisition (DIA), based on a quantitative proteomics technology, between the Δ*AHA_2124* strain and wild type, and there were 178 DAPs including 80 proteins up-regulated and 98 proteins down-regulated. The bioinformatics analysis showed that the deletion of gene *AHA_2124* led to some changes in the abundance of proteins related to multiple biological processes, such as translation, peptide transport, and oxidation and reduction. These results provided a theoretical basis for better exploring the regulatory mechanism of the MarR family transcriptional regulators of *Aeromonas hydrophila* on bacterial physiological functions.

## 1. Introduction

*Aeromonas hydrophila* is a major pathogen with human–animal–fish comorbidity [1] which is ubiquitous in the aquatic environment and predominantly pathogenic to fish, crabs, salmon, a variety of aquatic animals, and even terrestrial animals and humans [2,3,4,5]. *Aeromonas hydrophila*, belonging to the motile aeromonads of the genus *Aeromonas*, can cause motile *Aeromonas* septicemia (MAS), biological eruptions, sepsis, gastroenteritis, and necrotizing fasciitis when it infects fish, acting as an opportunistic pathogen, which then leads to serious economic losses worldwide [1,6,7,8]. This bacterium can rapidly adapt to various environmental conditions through a variety of molecular regulatory systems developed during its long evolutionary process [4,9]. Furthermore, the invasion and infection of *Aeromonas hydrophila* into the host need to be precisely regulated by a variety of transcriptional regulators which play major roles in many important biological processes such as cell division, virulence, quorum sensing, and stress resistance in prokaryotes [10,11,12].

The multiple antibiotic resistance regulator (MarR) protein family is an obligate homodimer widely found in bacteria and archaea [13]. The members of the MarR family are crucial to the survival of pathogenic bacteria, particularly for highly antibiotic-resistant pathogens [4,14]. Besides the role of antibiotic resistance, MarR family proteins also are multifunctional and play important roles in diverse biological functions. For example, HcaR, a member of the MarR family in *Acinetobacter*, controls the expression of HCA operons and has an effect on proteins catabolized by hydroxycinnamic acid derivatives [4]. CouR regulates the gene *cou*, responsible for p-hydroxycinnamic acid catabolism, in the soil bacterium *Haematococcus joecii* [13]. Moreover, several MarR family proteins, such as SlyA and PEC, are reported to play major roles in regulating the virulence genes of pathogens [4]. However, their effect and intrinsic regulatory mechanisms in aquatic pathogenic bacteria *Aeromonas hydrophila* have been largely unknown until now.

In the present study, we determined the physiological phenotypes of a gene deletion strain, and found the gene *AHA_2124*, encoding a MarR family protein, in *Aeromonas hydrophila*, significantly affected several phenotypes such as hemolytic activity, extracellular protease activity, and motility. We then compared the differences of the protein abundances between the deletion strain (Δ*AHA_2124*) and the wild type (WT) by using a quantitative proteomics method. Finally, the selected altered proteins in Δ*AHA_2124* were validated by Western blotting, and the effect of *AHA_2124* deletion on biological processes were analyzed using bioinformatics. In general, our results provided evidence for the better understanding of the biological functions and regulatory mechanism of MarR-type transcriptional regulators in *Aeromonas hydrophila*.

## 2. Materials and Methods

### 2.1. Bacteria Strains, Plasmids and Cultivation

*Aeromonas hydrophila* ATCC 7966 as the wild-type strain (WT), *Escherichia coli* S-17, and plasmids pRE112 were stored in our laboratory. Luria–Bertani (LB) media containing tryptone 10 g/L, yeast extract 5 g/L, and NaCl 10 g/L were used to culture the strains, which were incubated at 200 rpm at 30 °C for *Aeromonas hydrophila* or 37 °C for *E. coli*. A single clone of WT was added to 5 mL LB medium and cultured overnight at 30 °C at 200 rpm for 16 h. The overnight bacteria were transferred into LB media at a ratio of 1:100 (v:v) and then incubated to an OD_600nm_ of about 1.0 under 30 °C, at 200 rpm. Then, the bacteria were centrifuged for 1 min at 12,000 rpm and washed three times with phosphate-buffered saline (PBS) (pH = 7.4), before collecting the bacterial cells.

### 2.2. Construction of the Aeromonas hydrophila AHA_2124 Deletion Mutant

To construct Δ*AHA_2124* mutant strain using homologous recombination as in previous studies [15], the upstream (primers:P1/P2) and downstream (primers:P3/P4) sequences (Table 1) of *AHA_2124* were amplified by PCR. The two DNA fragments were fused into the pRE112 plasmid after double enzyme digestion (enzyme digestion sites *XbaI* and *SacI*) using Clone Express^®^ II One Step Cloning Kit (Vazyme Biotech Co., Ltd., Nanjing, China) to obtain the recombinant plasmid. The recombinant plasmid was transformed into *E. coil* Mc1061 competent cells. The plasmid was extracted and transformed into *E. coli* S17 competent cells after picking out the positive clones from the LB plate with 30 μg/mL chloramphenicol. Subsequently, the first homologous recombination was performed after the conjugation of *E. coli* S17 and WT at a ratio of 4:1 (*v/v*), and then the mixture was plated on an LB agar plate with 30 μg/mL chloramphenicol and 10 μg/mL ampicillin. The positive clones were cultured in the LB media for 16 h at 30 °C after being picked out from the LB agar plate (30 μg/mL chloramphenicol and 10 μg/mL ampicillin). Then, a single clone was picked out after being spread on an LB agar plate with 20% sucrose and cultured in LB medium. The bacteria were spread on the LB agar plate with 30 μg/mL chloramphenicol. The positive clones should not be alive on the last plate. Furthermore, the positive colonies were confirmed using PCR with primer pairs P5/P6 and P7/P8 (Table 1), and those DNA fragments could be amplified using P7/P8 and could not be amplified using P5/P6. The successful knockout strains were stored at −80 °C for further use.

### 2.3. Measurement of Hemolytic and Extracellular Proteolytic Activity

Hemolytic activity was tested on LB agar media supplemented with sheep blood (5% *v/v*), as previously described [16]. Briefly, 10 μL bacteria solution (OD_600nm_ = 1.0) was spotted into the sheep blood plate and incubated at 30 °C for 16 h. The extracellular proteolytic activity was tested on the plate containing skim milk (1% *v/v*), as previously described [17]. Briefly, 5 μL bacteria solution (OD_600nm_ = 1.0) was dropped on a perforated skim milk plate and incubated at 30 °C for 16 h. The diameters of the hydrolysis ring and hemolytic circle were recorded to determine the extracellular proteases and hemolytic activity, respectively. Every measurement was repeated independently in triplicate.

### 2.4. Bacterial Swimming and Swarming Motility Assays

The single colonies were stabbed into the swimming agar (0.3% *v/v*) plates and the swarming agar (0.5% *v/v*), and incubated at 30 °C for 8 h (swimming) or 12 h (swarming), respectively. The motility abilities were assessed by the migration of bacteria from the initial inoculation point. The experiments were detected independently in triplicate.

### 2.5. Protein Sample Preparation

The overnight bacteria were transferred to 20 mL LB culture media at a ratio of 1:100 (*v/v*) and cultured at 30 °C, 200 rpm, until OD_600nm_ = 1.0. After centrifuging at 5000 g for 10 min and discarding the supernatant, the bacteria were washed with PBS three times, then 0.5 mL of cell lysate was added (8 M Urea in 100 mM Tris–HCl pH 8.5). The bacterial cells were ultrasonically broken until the solution was clear, then centrifuged at 4 °C 18,000× *g* for 15 min. The supernatant was collected, and the concentration of the protein was determined with the Bradford kit. Finally, about 50 μg protein sample was reduced, alkylated, and digested using typsin to peptides according to the FASP (filter-aided sample preparation) method [18]. The digested peptides were desalted by a C18 desalt column (Waters Inc., Milford, MA, USA) and then dried using a CentriVap Concentrator (Labconco Inc., Kansas City, MO, USA).

### 2.6. DIA-Based LC-MS/MS

The desalted peptide samples obtained using enzymatic hydrolysis were dissolved in acetonitrile in 0.1% formic acid. A total of 100 μg of peptides from each sample were mixed and separated into 10 fractions using a RIGOL L-3000 HPLC system (Puyuanjingdian Science and Technology, Ltd., Beijing, China), as previously described [19]. DDA (data-dependent acquisition) and DIA (data-independent acquisition) analyses were both performed on an Orbitrap Fusion Lumos mass spectrometer (Thermo Scientific Inc., Waltham, MA, USA), equipped with an EASY-nLC 1200 system (Thermo Scientific Inc., Waltham, MA, USA). A total of 2 µg peptides containing iRT standard peptide (Biognosys, Schlieren, Switzerland) were loaded onto a 100 μm I.D. × 2 cm C18 trap column at a maximum pressure of 280 bar with 12 μL solvent A (0.1% formic acid in water, then separated on 150 μm I.D. × 25 cm column (C18, 1.9 μm, 120 Å, Dr. Maisch GmbH). The gradient, with a flow rate of 600 nL/min, was set as follows: 6–12% solvent B (80% ACN, 0.1 FA%) for 18 min, 12–20% solvent B for 59 min, 20–32% solvent B for 32 min, 32–80% solvent B for 1 min. Data were acquired with full scans (*m*/*z* 300–1400), acquired using an Orbitrap mass analyzer at a mass resolution of 120,000 at *m*/*z* 200. The top twenty precursor ions were selected for fragmentation in the HCD cell at normalized collision energy of 32%, and then fragment ions were transferred into the Orbitrap analyzer operating at a resolution of 30,000 at *m*/*z* 200. The automatic gain control (AGC) was set to 5e5 for full MS and 5e4 for MS/MS, with maximum ion injection times of 50 and 54 ms, respectively.

Each sample peptide was scanned using DIA analysis under the same LC system conditions as used for the mass spectrometry described above. For DIA analysis, the full scan was set at a resolution of 120,000, over an *m*/*z* range of 300 to 1400; followed by DIA scans with resolution 30,000; NCE:32%; AGC target: 5e5, and maximal injection time: 60 ms. A total of 45 variable DIA windows were set for DIA acquisition, ranging from 350 to 1400 *m*/*z*. 

The identification and quantification of the proteins were both finished by Spectronaut 18.0 (Biognosys, Schlieren, Switzerland) with default setting. Firstly, the DDA raw files were searched in Spectronaut pulsar against the *Aeromonas hydrophila* ATCC 7966 database (4121 entries, downloaded on 15 July 2019 from Uniprot) to generate a spectral library using the BGS factory setting. Peptides FDR\PSMs FDR\Proteins FDR were all set as 1%. The best 3–6 fragments per peptides were chosen for the spectral library. Then, DIA data were input into the software for protein quantification. The main parameter, iRT regression type, was set as local (non-linear) regression. All results were filtered by a Q value cutoff of 0.01 (corresponds to a FDR of 1%). P-value estimator was performed using the kernel density estimator. The area was used for quantification. Every peptide contained at least 3 fragment ions. The protein, with the difference of protein abundance ratio >1.5 times between Δ*AHA_2124* and WT, and the *p* value < 0.05 of Student’s *t* test between three biological repetitions of each sample, was selected as the differentially abundant protein (DAP). 

### 2.7. Western Blot

DAPs were selected to carry out Western blot analysis, as previously described [20]. Firstly, the protein samples were separated by sodium dodecyl sulfate polyacrylamide gel electrophoresis (SDS-PAGE), and then transferred to a polyvinylidene fluoride (PVDF) membrane using a BIO-RAD semi-dry transfer instrument (95 V, 35 min). Then, the PVDF membrane was blocked by PBST for 5 min, and incubated with home-made prime antibodies at room temperature for 2 h. After washing 5 times with PBST, the membranes were incubated with the secondary antibodies at room temperature for 1 h, and washed by PBST 5 times. Finally, the PVDF membranes were detected with a western ECL substrate (Bio-Rad Inc., Hercules, CA, USA) and the signal was displayed on the ChemiDoc MP imaging system (Bio-Rad Inc., Hercules, CA, USA).

### 2.8. Bioinformatics Analysis

Gene Ontology (GO) analysis of DAPs was performed using the online DAVID software (https://david.ncifcrf.gov/ (accessed on 5 September 2023)) [21]. The protein–protein interaction (PPI) network was constructed using The Search Tool for the Retrieval of Interacting Genes (STRING) online software version 11.5 (https://string-db.org (accessed on 5 September 2023)) and visualized using Cyctoscape 3.10.0 [22]. Module analysis was conducted using the molecular complex detection (MCODE) plug-in, and hub proteins were ranked using the CytoHubba plugin in Cytoscape software with default settings [23,24].

## 3. Results

### 3.1. Construction and Phenotype Characterization of the AHA_2124 Deletion Strain in Aeromonas hydrophila 

**Figure 1:**
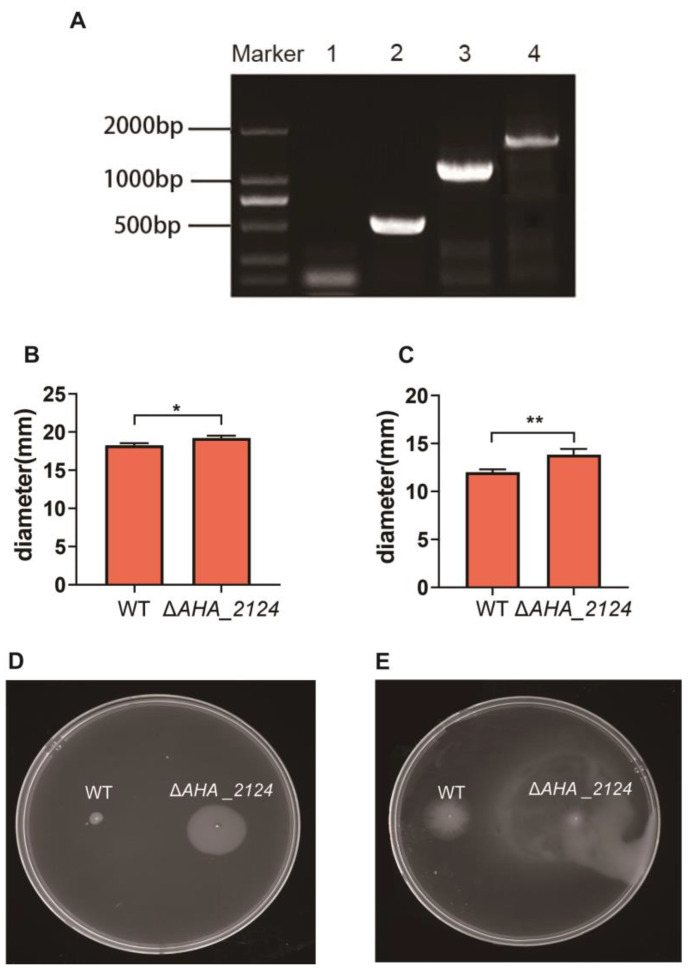
Construction and the phenotype assays of Δ*AHA _2124* in *Aeromonas hydrophila***.** (**A**) PCR amplification confirmed the construction of Δ*AHA_2124*. M, DL 2000 bp DNA marker; lane 1, Δ*AHA_2124*-P5/P6; lane 2, WT-P5/P6(567 bp); lane 3, Δ*AHA_2124*-P7/P8 (1279 bp); lane 4, WT-P7/P8 (1846 bp). (**B**) Extracellular protease activity of WT and Δ*AHA_2124*. Data are the means of three independent experiments and presented as means ± SD. * *p* < 0.05. (**C**) Hemolytic activity of WT and Δ*AHA_2124*. Data are the means of three independent experiments and presented as means ± SD. ** *p* < 0.01. (**D**) Swarming motility analysis of WT and Δ*AHA_2124*. (**E**) Swimming motility analysis of WT and Δ*AHA_2124*.

The mutant with the *AHA_2124* gene deletion was constructed according to the designed primers as shown in Table 1. The PCR identification showed that the *AHA_2124* mutant strain (Δ*AHA_2124*) was successfully constructed (Figure 1A). The assays of the phenotypes showed that Δ*AHA_2124* had significantly increased extracellular protease activity (Figure 1B) and hemolytic activity (Figure 1C) when compared with the wild type (WT) strain. Moreover, the deletion of *AHA_2124* significantly improved the swarming and swimming abilities as well (Figure 1D,E). Taken together, these results indicated that the deletion of the *AHA_2124* gene significantly affected the bacterial physiological phenotypes and may negatively regulate the pathogenicity of *Aeromonas hydrophila*.

### 3.2. Proteomics Analysis of Protein Abundance between ΔAHA_2124 and Aeromonas hydrophila WT Strain

**Figure 2:**
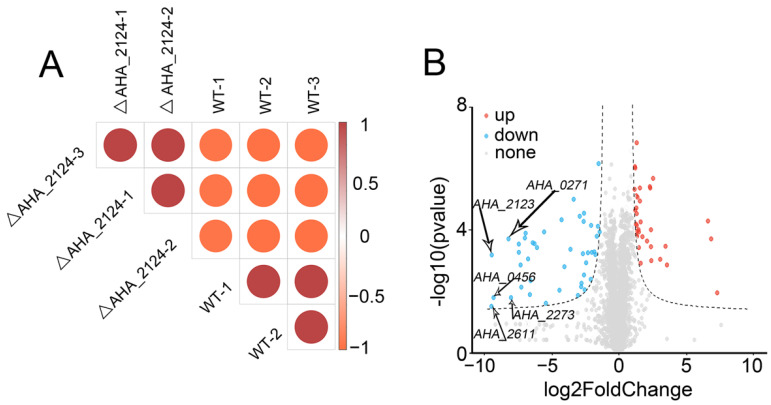
Quantitative proteomics analysis between Δ*AHA_2124* and WT strain. (**A**) The correlation analysis among all samples of Δ*AHA_2124* and WT by calculating correlation coefficient; (**B**) volcano plot visualization of differential expression in analysis between Δ*AHA_2124* and WT strain. Each point represents one gene; the orange dots represent up-regulated genes; the blue dots represent down-regulated genes.

In this study, quantitative proteomics techniques were used to analyze the DAPs in Δ*AHA_2124* and WT *Aeromonas hydrophila*. A total of 2654 proteins (shown in the Supplementary Material Table S1), accounting for 64.37% of the total protein content of WT *Aeromonas hydrophila*, were identified with double criteria (unique peptide in each protein match number ≥2, with proteins and peptides FDR < 1%). Furthermore, the correlation analysis among each repeated sample was shown in Figure 2A, and the correlation coefficients (R > 0.95) indicated a good reproducibility for further analysis. The following volcano plot displayed that a total of 178 DAPs were identified, including 80 proteins up-regulated and 98 proteins down-regulated in Δ*AHA_2124* when comparted to WT strain (Figure 2B). Some DAPs (the top 12 of increased abundance and top 12 of decreased abundance) were selected, and the information on these proteins are shown in Table 2.

### 3.3. Validation of Proteomics Results

**Figure 3:**
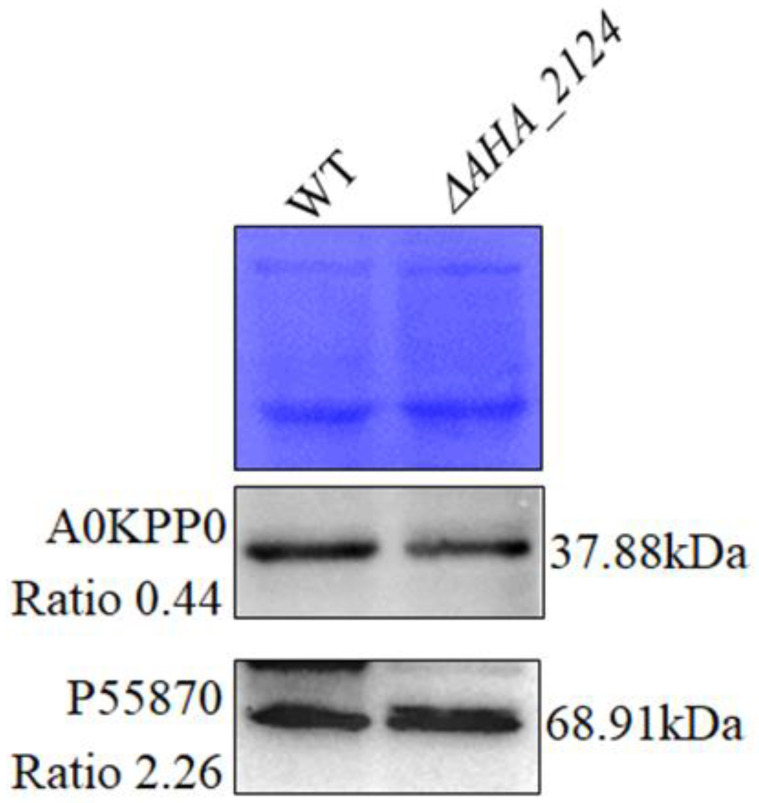
The validation of proteomics analysis results using Western blot. Coomassie R-350 staining of the membrane indicated equal loading of protein samples on the top, with Western blot results of two selected proteins shown on the bottom; the ratio value is from the results of quantitative proteomics analysis.

To verify the reliability of the quantitative proteomics results, two DAPs were selected and their expression in the Δ*AHA_2124* and WT strains on the protein level were determined using a Western blot. The WB original images of the two proteins are shown in Supplementary Figures S1 and S2, and the intensity ratio of the target proteins are shown in Supplementary Figure S3. As shown in Figure 3, when compared with the WT strain, the expression of A0KPP0 was down-regulated, and the expression of Ahh1 (P55870) was up-regulated, which were consistent with the quantitative proteomics results, confirming the good reliability and stability of the MS data.

### 3.4. Functional Analysis of Differentially Abundant Proteins between Δ AHA_2124 and WT Strain

**Figure 4:**
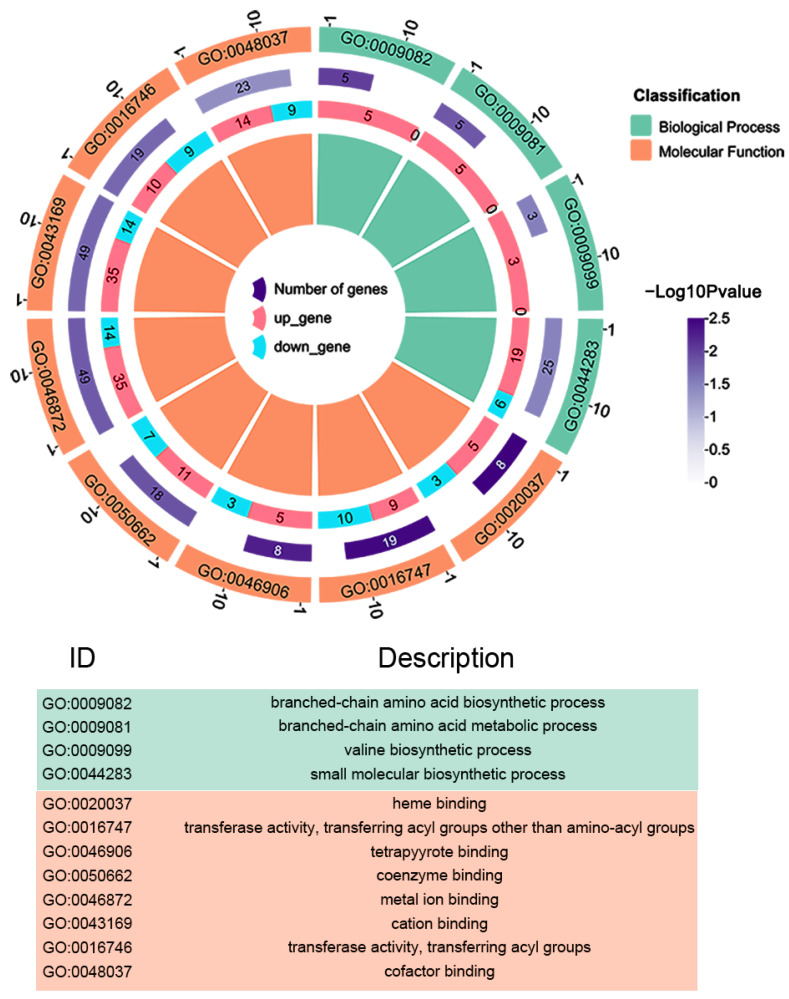
Bioinformatic analysis of DAPs between Δ*AHA_2124* and WT strain. GO enrichment analysis of biological processes (BPs), cellular component (CCs) and molecular function (MFs) in differential expression proteins between Δ*AHA_2124* and WT. The colors of the table below correspond to the above figure.

To further explore the effect of *AHA_2124* on *Aeromonas hydrophila*, the GO enrichment analysis of the DAPs was performed using DAVID software (Figure 4). The results showed that the DAPs were mainly involved in several important biological processes (BPs), cell components (CCs), and molecular functions (MFs) of the Δ*AHA_2124* strain and WT *Aeromonas hydrophila*. In the biological processes classification, 25 DAPs were mainly enriched in small molecular biosynthetic processes (GO:0044283), 5 DAPs in branched-chain amino acid biosynthetic processes (GO:0009082), and 5 DAPs in branched-chain amino acid metabolic processes (GO:0009081). Furthermore, GO enrichment of the molecular functions (MFs) category of the DAPs showed that 49 DAPs mainly functioned in cation binding (GO:0043169), 49 DAPs in metal ion binding (GO:0046872), 23 DAPs in cofactor binding (GO:0048037), 19 DAPs in transferase activity (GO:0016747), and 19 DAPs in transferase activity, transferring acyl groups (GO:0016746).

### 3.5. Protein–Protein Interaction Network of DAPs between WT and ΔAHA_2124 Strain

**Figure 5:**
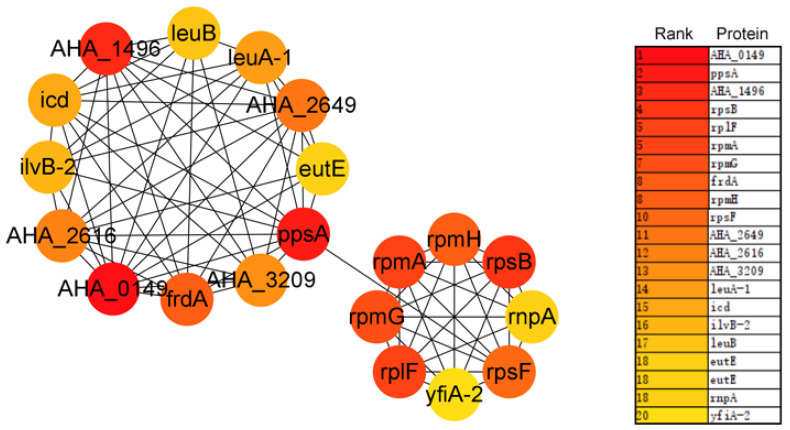
The protein–protein interaction (PPI) network of top 20 hub proteins between Δ*AHA_2124* and WT strain.

**Figure 6:**
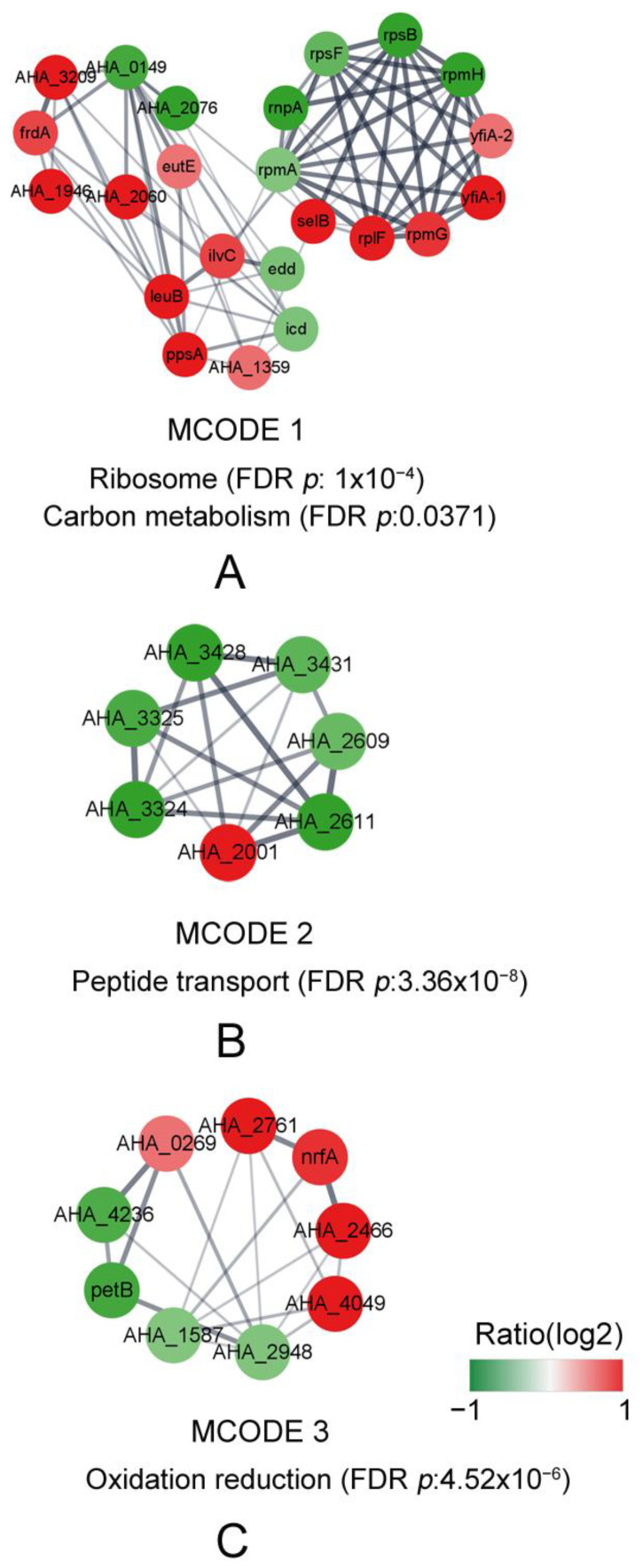
The module analysis of DAPs between Δ*AHA_2124* and WT strain. (**A**) Module 1 contained 23 genes nodes and 73 edges, MCODE score = 6.636. MCODE 1. (**B**) Module 2 contained 7 nodes and 16 edges, MCODE score = 5.33. (**C**) Module 3 contained 9 nodes and 18 edges, MCODE score = 4.5. Green represents downregulated genes and red represents up-regulated genes. DAPs: differentially abundant proteins; MCODE: molecular complex detection.

The protein–protein interaction (PPI) network of the DAPs was analyzed by the STRING online software; the hub proteins and module were analyzed by Cytoscape with MCODE and CytoHubba plugins, respectively. As is shown in Figure 5, the top 20 hub proteins are A0KEL7 (gene ID: *AHA_0149*), A0KID3 (*AHA_1496*), A0KLH4 (*AHA_2616*), A0KLK7 (*AHA_2649*), A0KN49 (*AHA_3209*), EutE, FrdA, Icd, IlvB-2, LeuA-1, LeuB, PpsA, RnpA, RplF, RpmA, RpmG, RpmH, RpsB, RpsF, and YfiA-1, and most of them were involved in ribosome function, the biosynthesis of secondary metabolites, and microbial metabolism in diverse environments. The top three modules were further analyzed with KEGG or GO enrichment. Of these modules, six ribosome-related proteins (RpmG, RplF, RpsF, RpmA, RpsB, and RpmH) and five carbon metabolism proteins (gene ID *idd*, *icd*, *ppsA*, *frdA*, and *AHA_3209*) were significantly enriched in MCODE 1 (Figure 6A). A total of seven peptide transport proteins (*AHA_2001*, *AHA_2609*, *AHA_2611*, *AHA_3324*, *AHA_3325*, *AHA_3428*, and *AHA_3431*) were significantly enriched and most of them showed decreased abundances in MCODE 2, except for A0KJT1 (*AHA_2001*) (Figure 6B), and the expression of nine oxidation reduction process-related proteins were enriched in MCODE 3 (Figure 6C).

## 4. Discussion

The MarR proteins, a sub-group of winged helix-turn-helix (wHTH) DNA-binding proteins, regulate the expression of genes and play important roles in the regulation of diverse biological functions, such as resistance to multiple antibiotics, metabolism, biofilm formation, motility, and virulence in different pathogenic bacteria [25,26,27,28]. There are six members of the MarR family annotated in *Aeromonas hydrophila* ATCC 7966, encoded by the genes *AHA_1240*, *AHA_2124*, *AHA_3609*, *AHA_1539*, *AHA_3721*, *AHA_0734*, respectively.

In order to explore the effect of the gene *AHA_2124* on the behavior of *Aeromonas hydrophila*, we constructed a *ΔAHA_2124* mutant strain in *Aeromonas hydrophila* ATCC 7966, and found that the deletion of *AHA_2124* led to a significant increase in the bacterial hemolytic and extracellular protease activities. It has been reported that the extracellular products secreted by *Aeromonas hydrophila* contain a variety of enzymes and hemolysins that possess hemolytic, cytotoxic, cytolytic, and protease, and could lead to infection in fishes [29,30,31]. Furthermore, the MarR family members in pathogenesis play an important role in controlling the virulence-associated traits and the virulence-regulated gene [32,33,34]. Thus, the results of the physiological phenotypes indicated that *AHA_2124* may negatively regulate the virulence of *Aeromonas hydrophila*.

Based on the proteomics analysis, our results showed that 178 DAPs were identified in the Δ*AHA_2124* strain and WT *Aeromonas hydrophila*. From these DAPs, we selected two proteins (A0KPP0 and P55870) to validate using Western blot, and the results were consistent with the quantitative proteomics results. The A0KPP0 protein, an iron-related protein, was down-regulated after the deletion of the gene *AHA_2124*. Iron is an essential element for living cells, and is also a component of essential biological processes, such as DNA replication and energy production in both microbial pathogens and animals [35]. Hemolysin ahh1 (P55870), being a virulence factor, is regulated by *Uvry* and is also a type of pore-forming toxin [36]. Interestingly, hemolysin Ahh1 was up-regulated after the deletion of the gene *AHA_2124* in the present study, in accordance with the result of hemolytic activity. The validation of these two proteins was to prove the stability and reliability of the MS data for further analysis.

The deletion of *AHA_2124* also significantly enhanced the bacterial motility in this study. Motile bacteria can respond more quickly to environments and obtain a more favorable ecological niche, and that largely depends on the bacterial cilium and extracellular flagellum. Motility plays a crucial role in bacterial adaptation and is known to be related to the virulence of a number of pathogens [37,38]. It was reported that MarR-type transcriptional regulators are involved in the regulation of bacterial motility capability [39]. For example, the deletion of the transcription factor SlyA of the MarR family significantly enhances the motility of the *EC1* strain of *Phytophthora zea* [40]. As another example, *papX* or *focX* (encoding a MarR transcription factor) was reported to negatively regulate the movement of uropathogenic *E. coli* (UPEC) [39]. In this study, many flagellar or fimbrial-related proteins were altered in the *ΔAHA_2124* strain. There were three up-regulated proteins (putative flagellar hook-length control protein FliK, flagellin A0KIY4, and flagellar hook-associated protein 2 fliD) and five down-regulated proteins (Msha pilin protein MshC, flagellar export protein FliJ, fimbrial proteins A0KFN0 and A0KFN1, and fimbrial chaperone protein A0KFM9). These results indicated the important role of gene *AHA_2124* on the regulation of bacterial migration ability in *Aeromonas hydrophila*.

Additionally, many transcriptional regulators were changed to a certain extent after the deletion of *AHA_2124* according to the proteomics results, and the most abundant of the transcriptional regulators belongs to the LysR family (twenty-two), TetR family (eight), GntR family (five), MerR family (three), AsnC family (two), LuxR family (three), AraC family (two), and LitR family (one). The LysR family of regulators is the most abundant type of transcriptional regulator in prokaryotes and is a well-known family involved in diverse physiological functions [41]. Similarly to the MarR families, the AsnC family proteins also have the H-T-H (helix-turn-helix) domains and are related to amino acid biosynthesis [42,43]. In our previous studies, the overexpression of four putative LysR family proteins (A0KIU1, A0KJ82, A0KPK0, and A0KQ63) led to increased sensitivity to several antibiotics, and the LysR family proteins play an important role in the antibiotic resistance of *A. hydrophila* [44]. Furthermore, it was reported that *yeeY*, a LysR-type transcriptional regulator (LTTR), negatively regulated furazolidone resistance according to the proteomics analysis [45]. Zhang et al. reported that ORF02889, encoded by *orf02889* (an AraC-like transcription factor), was involved in the regulatory mechanism for the virulence of *Aeromonas hydrophila* [46]. Thus, these transcriptional regulators described previously were all affected by the deletion of *AHA_2124* in this study, indicating the important role of *AHA_2124* in some physiological processes of *Aeromonas hydrophila*.

In the PPI network construction based on the STRING online software, the top 20 hub proteins (Figure 5) were identified when studying the large-scale protein network, such as RpmA, RpmG, RpmH, RpsB, RpsF, RnpA, RplF, YfiA-1, FrdA, Icd, LeuB, and so on. Moreover, most of the proteins were also recognized as hubs in MCODE1 (Figure 6A), which are mainly related to ribosome and carbon metabolism, being consistent with the results of the top 20 hub proteins. RpmA is a RTX-family protein which is involved in aggregation and biofilm formation [47] and is described in enteric bacteria which comprise one component of the flagellar motor [38]. Suigo et al. reported that RnpA may be one of the main players in the bacterial RNA degradation and processing machinery which are essential processes for bacterial viability in *Staphylococcus aureus* [48], and RnpA seems to be involved in cellular mRNA degradation as well as a second RNA-metabolic process [49]. In MCODE 2 (Figure 6B), seven enriched proteins were related to peptide transport and were decreased, except for A0KJT1. The proteins involved in peptide transport play important roles in the conservation of peptide-bound amino nitrogen from the environment [50,51]. In addition, nine oxidation–reduction process-related proteins were enriched in MCODE 3 (Figure 6C). The results of the PPI analysis indicated that most DAPs related to ribosome and carbon metabolism, peptide transport, and the oxidation–reduction process were changed when the gene *AHA_2124* was deleted. It suggested that the protein of the MarR family encoded by gene *AHA_2124* is involved in lots of biological processes.

## 5. Conclusions

In this study, we have studied the bacterial phonotypes of a MarR-type transcriptional regulator *AHA_2124*, and found that the deletion of *AHA_2124* affected several common physiologic functions, such as extracellular protease activity, hemolytic activity, and motility. The following proteomics analysis between the Δ*AHA_2124* and WT strain reveal that this MarR-type transcriptional regulator is involved in many important biological processes. In general, our research may contribute to the understanding of the biological function and molecular regulatory mechanisms of the transcriptional regulators of the MarR family of *Aeromonas hydrophila* and provide insight on the prevention and control of this pathogen in the future.

## Figures and Tables

**Table 1:** The primers used for the construction of the *AHA_2124* deletion mutant and verification in *Aeromonas hydrophila*.

Primer Name	Primer Sequence (5′→3′)
P1	CGATCCCAAGCTTCTTCTAGACCGACTGCCTGCTGCGTC
P2	TCAGGTCAGAGAAATGTGCGGTTGCG
P3	CGCACATTTCTCTGACCTGAACAACTTTGCCACCA
P4	CATGAATTCCCGGGAGAGCTCCTGCCGACCATCAGGCAG
P5	GGATCTTCCAGAGATGTGCATGTTCAGACAAACGGC
P6	CTGCCGTTCGACGATTCATGAGGGTCGCTCGCTAT
P7	GGATCTTCCAGAGATGAGCCGCTGTTCGACGCC
P8	CTGCCGTTCGACGATTGCCCAGGCCAAGGGCAT

**Table 2:** Top 12 DAPs of increased abundance and top 12 DAPs of decreased abundance between Δ*AHA_2124* and WT strain.

Accession	Gene	Descriptions	*p*-Value	log2 (Δ*AHA_2124*/WT)
A0KI69	*AHA_1431*	Pts system, fructose-specific iiabc component	1.11 × 10^−23^	10.11
A0KFI7	*AHA_0480*	Uncharacterized protein	6.83 × 10^−10^	7.95
A0KK36	*polB*	DNA polymerase	6.49 × 10^−9^	7.64
A0KLW8	*AHA_2761*	4Fe-4S binding domain protein	1.96 × 10^−14^	7.18
A0KEP9	*ubiA*	4-hydroxybenzoate octaprenyltransferase	9.89 × 10^−15^	6.94
A0KQJ3	*AHA_4120*	Probable nitrite transporter	2.23 × 10^−11^	5.90
A0KFS4	*AHA_0567*	Heat shock protein 15	7.26 × 10^−8^	3.78
A0KK31	*lpxM*	Lipid A biosynthesis myristoyltransferase	2.49 × 10^−6^	3.77
A0KM07	*AHA_2803*	Uncharacterized protein	7.30 × 10^−4^	3.67
A0KLP4	*AHA_2687*	Microbial serine proteinase	2.54 × 10^−3^	3.27
A0KQ31	*AHA_3948*	Uncharacterized protein	1.55 × 10^−9^	2.72
A0KL71	*selB*	Selenocysteine-specific translation elongation factor	1.49 × 10^−6^	2.58
A0KL58	*fdhA*	Formate dehydrogenase, alpha subunit, selenocysteine-containing	4.54 × 10^−4^	2.55
A0KLH0	*AHA_2612*	Oligopeptide ABC transporter, permease protein OppB	1.80 × 10^−10^	−7.84
A0KKJ5	*AHA_2273*	Phospholipase, patatin family	7.18 × 10^−7^	−8.19
A0KEY3	*AHA_0271*	Uncharacterized protein	6.46 × 10^−13^	−8.39
A0KQT0	*AHA_4209*	Phosphate acyltransferase family protein	1.13 × 10^−5^	−8.55
A0KIW0	*AHA_1675*	Uncharacterized protein	3.20 × 10^−5^	−8.85
A0KLV6	*AHA_2749*	Putative membrane protein	6.70 × 10^−3^	−9.41
A0KFG4	*AHA_0456*	Acetyltransferase	2.07 × 10^−8^	−9.55
A0KK46	*AHA_2123*	Uncharacterized protein	2.83 × 10^−12^	−9.69
A0KLG9	*AHA_2611*	Oligopeptide ABC transporter, permease protein OppC	1.44 × 10^−9^	−9.72
A0KLT3	*AHA_2726*	Na+/H+ antiporter family protein	4.95 × 10^−5^	−12.40
A0KLQ5	*AHA_2698*	Diguanylate cyclase/phosphodiesterase domain 1	1.25 × 10^−11^	−13.02

## Data Availability

The mass spectrometry proteomics data have been deposited to the ProteomeXchange Consortium (http://proteomecentral.proteomexchange.org (accessed on 2 November 2023)) via the iProX partner repository with the dataset identifier PXD046625.

## Supplementary Materials

The following supporting information can be downloaded at https://www.mdpi.com/article/10.3390/biology12121473/s1. Table S1: The whole list of 2654 proteins based on MS data; Figure S1: The original WB image of the A0KPP0 protein; Figure S2: The original WB image of the P55870 protein; Figure S3: The intensity ratio of the target proteins in each group of WB results based on the analysis of ImageJ software.
