# Supplementary material for: Quantitative Proteomics Analysis Reveals the Effect of a MarR Family Transcriptional Regulator AHA_2124 on Aeromonas hydrophila
Source: Biology (Basel). 2023 Nov 28;12(12):1473. doi: 10.3390/biology12121473 (PMC10740729; doi:10.3390/biology12121473)
Supplement: Supplementary file 1 [file biology-12-01473-s001.zip › Supplementary Figures.pdf]

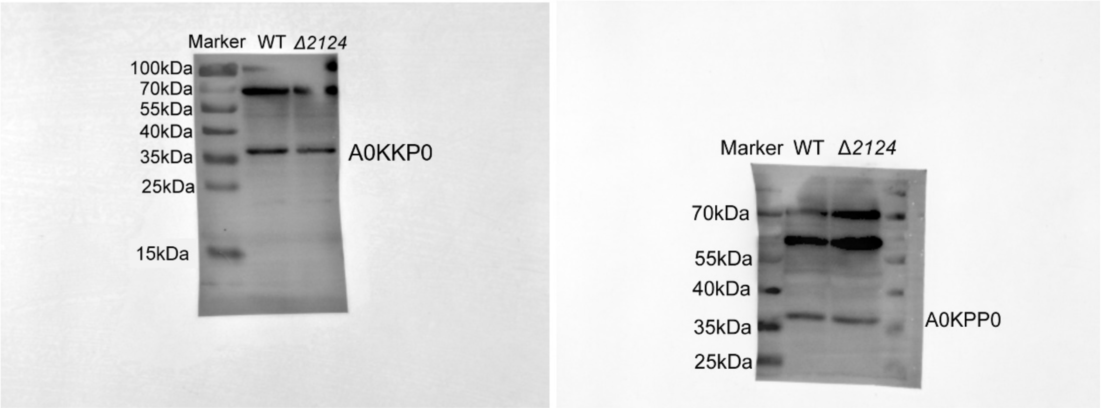

Figure S1 The original WB image of the A0KPP0 protein

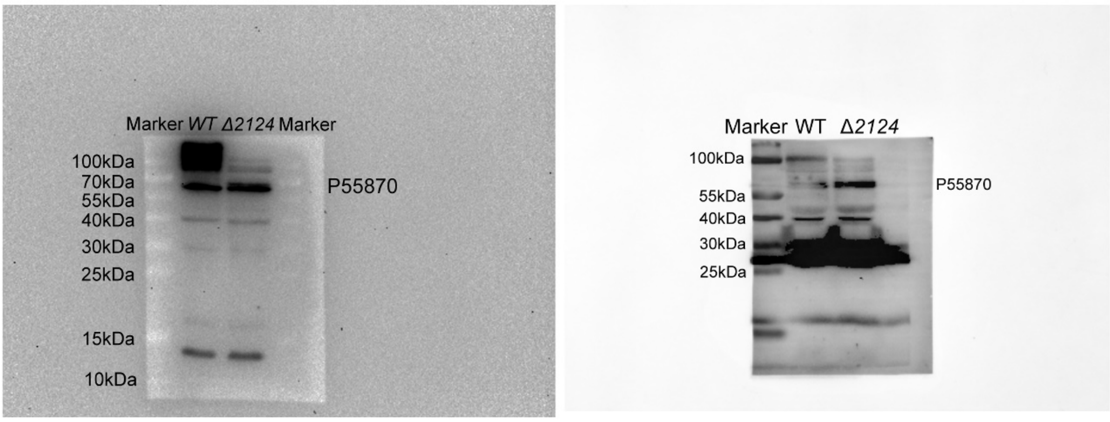

Figure S2 The original WB image of the P55870 protein

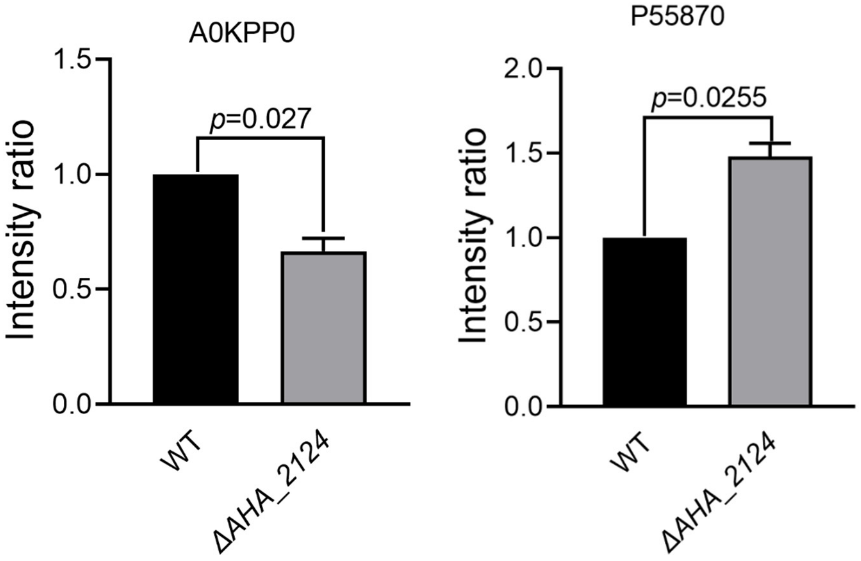

Figure S3 The intensity ratio of the target proteins in each group of WB results based on the analysis of ImageJ software
